# Profiles of PD-1, PD-L1, PD-L2 in Gastric Cancer and Their Relation with Mutation, Immune Infiltration, and Survival

**DOI:** 10.1155/2020/2496582

**Published:** 2020-06-06

**Authors:** Jingwei Liu, Hao Li, Liping Sun, Yuan Yuan, Chengzhong Xing

**Affiliations:** ^1^Tumor Etiology and Screening Department of Cancer Institute and General Surgery, The First Hospital of China Medical University, Shenyang 110001, China; ^2^Key Laboratory of Cancer Etiology and Prevention (China Medical University), Liaoning Provincial Education Department, Shenyang 110001, China; ^3^Key Laboratory of Gastrointestinal Cancer Etiology and Screening, Shenyang, 110001 Liaoning Province, China

## Abstract

**Background:**

Although multiple types of cancers demonstrated favorable outcome after immunotherapy of PD-1/PD-L1 blockade, the specific regulatory mechanism of PD genes in gastric cancer (GC) remains largely unknown.

**Materials and Methods:**

Expression of RNA, copy number variants, and clinical parameters of GC individuals from TCGA were analyzed. Coexpressed genes for PD-1, PD-L1, and PD-L2 were selected by correlation analysis and confirmed by STRING. Gene Ontology and KEGG pathway analyses were performed by clusterProfiler. The influence of PD-1/PD-L1/PD-L2 on immune cell infiltration was investigated by MCP-counter.

**Results:**

PD-L2 demonstrated significant relation with clinical stage of GC (*P* = 0.043). Survival analysis showed that PD-1 expression was correlated with better prognosis of GC patients (HR = 0.70, *P* = 0.031), but PD-L2 expression was related with worse survival (HR = 1.42, *P* = 0.032). Mutation of *PIK3CA* could alter the level of PD-1, PD-L1, and PD-L2 (*P* < 0.001), and *TP53* mutation demonstrated significant correlation with PD-L1 (*P* = 0.015) and PD-L2 (*P* = 0.014) expression. Enrichment analysis of PD-1/PD-L1/PD-L2 coexpressed genes indicated a biological process of mononuclear cell proliferation, leukocyte cell-cell adhesion, and lymphocyte activation as well as KEGG pathways including cell differentiation of Th1 and Th2, cell differentiation of Th17, and hematopoietic cell landscape. As for immune infiltration analysis, PD-1 was mainly related with cytotoxic lymphocytes and endothelial cells; PD-L1 were associated with monocytic lineage; PD-L2 showed significant correlation with myeloid dendritic cells.

**Conclusion:**

PD-1 expression showed association with better prognosis of GC, and PD-L2 expression was related with worse survival. Mutations of *PIK3CA* and *TP53* significantly correlated with PD-1/PD-L1/PD-L2 axis. PD-1/PD-L1/PD-L2 coexpressed genes demonstrated enrichment in mononuclear cell proliferation, leukocyte cell-cell adhesion, and lymphocyte activation as well as KEGG pathways including cell differentiation of Th1, Th2, and Th17.

## 1. Introduction

Gastric cancer (GC) is a refractory cancer in the human upper digestive system; the incidence and mortality of which remain relatively high all around the world [1, 2]. Although great progress has been made in the therapy of gastric cancer, a large amount of GC patients still suffer an unsatisfactory prognosis [3]. One of the most intractable challenges in clinical treatment of GC is that only a part of GC patients benefit from traditional chemical treatment strategy, indicating other elements which also affect the clinical outcome including human immune reaction [4, 5].

One of the most encouraging breakthroughs about cancer therapy in recent years is supposed to be the application of antibody for PD-1/PD-L1 in treatment of a series of cancers [6]. Up to now, multiple types of cancers demonstrated favorable outcome after immunotherapy of PD-1/PD-L1 blockade including lung cancer, melanoma, breast cancer, and renal cancer [7, 8]. Programmed death 1 (PD-1) and its ligands programmed death ligand 1 (PD-L1) and programmed death ligand 2 (PD-L2) serve as an immune checkpoint axis which can be utilized by cancer cells for immune escape from destruction by T cells [9, 10]. Specifically, PD-1, encoded by the *PDCD1* gene, interacts with corresponding ligands PD-L1 and PD-L2 to suppress T cell activation and make immune surveillance invalid [11, 12]. Emerging evidence concerning experimental investigation and clinical trials suggested promising application of PD-1/PD-L1 blockade in gastrointestinal malignancies. In a multicenter clinical trial of pembrolizumab treatment for PD-L1+ advanced GC patients, anti-PD-1 antibody pembrolizumab exerts acceptable toxicity status and a great antitumor effect [13]. Interferon gamma has been reported to increase PD-1 expression in gastric cancer cells via the JAK-signal transducer and activating transcription pathway [14]. As key component of bacterial infection, lipopolysaccharide (LPS) induced PD-L1 expression in GC cells by activating the NF-*κ*B pathway [15]. In addition, PD-L1 leads to apoptosis of T cells in GC cells, and T cells upregulate PD-L1 with the effect of IFN-*γ* [16].

As a powerful approach in therapy of various types of cancer, the PD-1 and PD-L1 blockade immunotherapy has benefit in many clinical individuals with malignant tumor including gastric cancer. Until now, however, the specific regulatory mechanisms of this novel immune pathway are still elusive. Therefore, we systematically investigated the expression data from TCGA in order to characterize the distributions of PD-1, PD-L1, and PD-L2 in relation to clinical parameters and survival of gastric cancer. Additionally, association of somatic mutation, immune cell infiltration, and other essential immune markers with the PD-1 axis was also analyzed to unravel the importance of PD-1 and its ligands in determination of human immune microenvironment status in gastric cancer.

## 2. Materials and Methods

### 2.1. Raw Data

The RNA expression, copy number variants, and clinical information of gastric cancer individuals of TCGA were obtained by UCSC XENA. Transcripts per million reads were used to assess the expression level of RNAs. Clinical information contained age, gender, stage, tumor recurrence, and survival.

### 2.2. Correlated Genes and Functional Enrichment

Using coexpression analysis, the correlated genes of PD-1, PD-L1, and PD-L2 were obtained. Genes of correlation coefficient *r* > 0.6 with PD-1/PD-L1/PDL-2 were selected as the candidate genes. After identification of the interaction genes, we used protein-protein interaction analysis to confirm the interactions among genes by STRING (https://string-db.org). The clusterProfiler method was then performed for the functional enrichment of Gene Ontology to interpret the interaction of the genes.

### 2.3. Association of Immune Factors with PD-1, PD-L1, and PD-L2

Immune cell infiltration has been widely reported to be implicated in multiple processes of cancer. MCP-counter R package was used to assess the infiltration of immune cells, which gives each individual an available score of CD3+ T cells, CD8+ T cells, cells originating from monocytes, NK cells, cytotoxic and B lymphocytes, myeloid dendritic cells, neutrophils, endothelial cells, and fibroblasts. Correlation was analyzed to explore the relation of PD genes with immune cell infiltration. In addition, the specific correlation of PD genes with key immune checkpoints was also investigated.

### 2.4. Statistical Analysis

Most statistical analysis of this research was conducted by use of R language including several online available packages. We used the rank sum test to detect PD gene expression difference in various groups. The relation of PD genes with immune cell infiltration and key immune factors was detected by the Spearman correlation. Kaplan-Meier analysis was conducted with the log-rank method to draw the survival curve of prognosis. Other R packages of ComplexHeatmap (17) as well as corrplot were also adopted when needed. The B-H method was conducted to limit the error of multiple comparisons. A *P* value < 0.05 means statistical significance in the present research.

## 3. Results

### 3.1. Expression of PD-1, PD-L1, PD-L2, and Clinical Parameters

**Figure 1:**
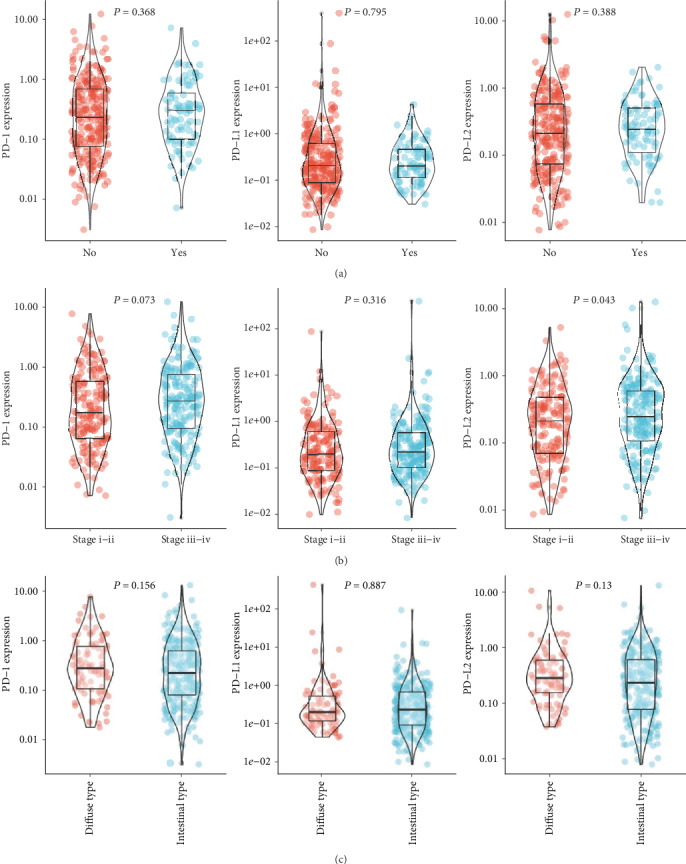


Based on the data of TCGA, we explored the relationship of PD gene expression with multiple clinical parameters. As shown in Figure 1(a), PD-1/PD-L1/PD-L2 demonstrated no significant association with recurrence of gastric cancer. In addition, PD-L2 was associated with clinical stage (*P* = 0.043), while no significant relation was observed for PD-1 (*P* = 0.073) and PD-L1 (*P* = 0.316) (Figure 1(b)). No significant difference of PD-1, PD-L1, or PD-L2 expression was observed between the diffuse type and intestinal type gastric cancer (Figure 1(c)).

**Figure 2:**
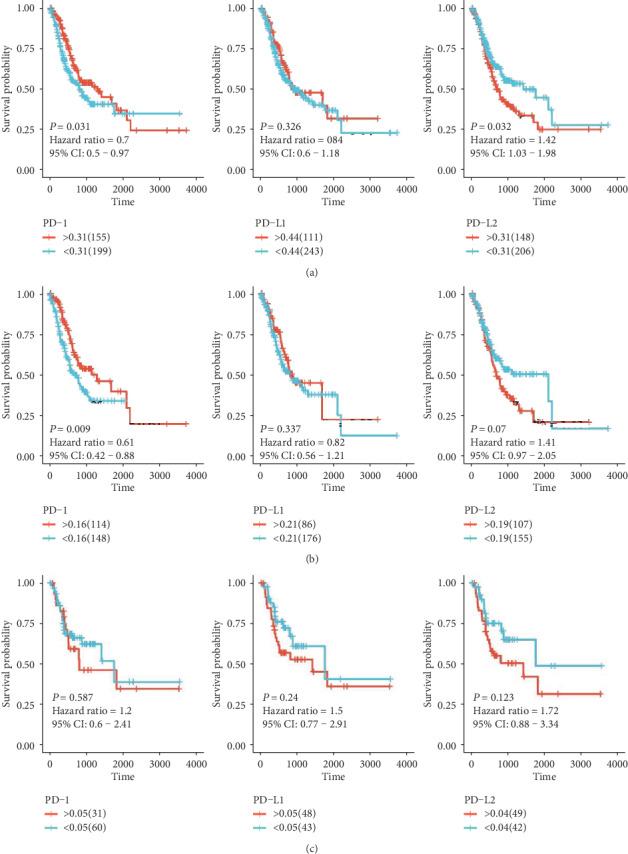


Survival analysis of GC patients showed that PD-1 expression was related with favorable survival of GC patients (HR = 0.70, 95%CI = 0.50 − 0.97, *P* = 0.031) (Figure 2(a)). On contrary, PD-L2 expression was significantly related with poor survival of GC (HR = 1.42, 95%CI = 1.03 − 1.98, *P* = 0.032). As for PD-L1, no significant relation was observed for GC prognosis (HR = 0.84, 95%CI = 0.60 − 1.18, *P* = 0.326). In the subgroup of diffuse type gastric cancer, PD-1 expression was associated with better prognosis (HR = 0.61, 95%CI = 0.42 − 0.88, *P* = 0.009) (Figure 2(b)). As for intestinal type gastric cancer, no significant association of PD-1, PD-L1, or PD-L2 with survival was found (Figure 2(c)).

### 3.2. Copy Number Variation and Mutation

**Figure 3:**
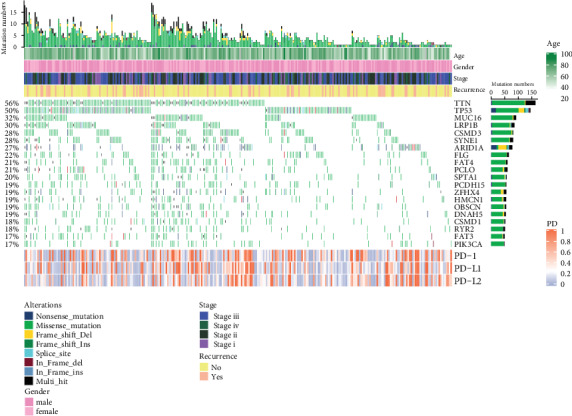


Copy number variants of 290 patients based on TCGA data were analyzed. A total of 20 mutations at the highest occurrence frequency were adopted and is visualized in Figure 3. PD gene expression showed no significant association with the entire mutation burden of each individual (*R* = 0.06/0.07/0.08, respectively). However, after differential expression analysis, PIK3A mutations might be associated with the expression levels of PD-1, PD-L1, and PD-L2 (all *P* < 0.001). And TP53 showed significant association with expression of PD-L1 (*P* = 0.015) and PD-L2 (*P* = 0.014) (Table 1).

### 3.3. Correlated Genes of PD-1, PD-L1, and PD-L2

**Figure 4:**
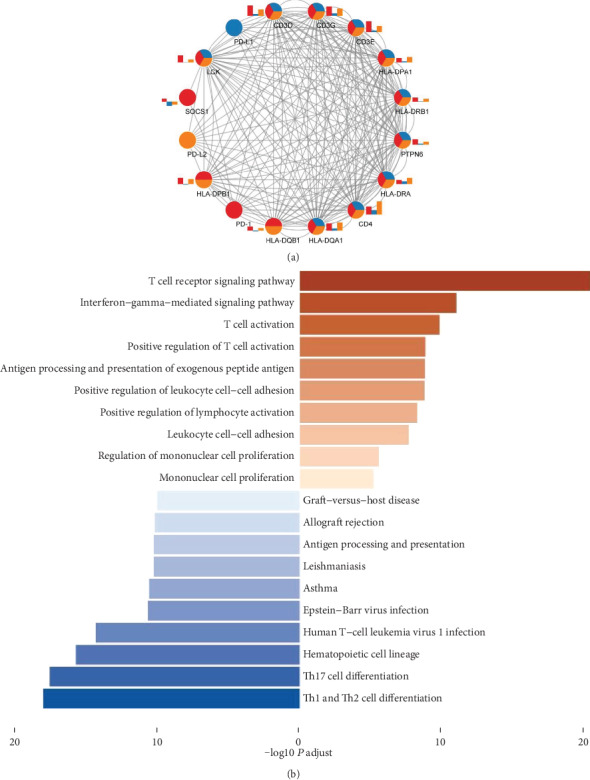


After coexpression analysis, we finally obtained 831 PD-1 correlated genes, 1162 PD-L1 correlated genes, and 1997 genes interacting with PD-L2. Then, we verified the two module interaction in STRING datasets (Figure 4(a)). After verification, PD-L1 interacted with 10 genes; PD-L2 interacted with 12 genes while PD-1 showed coexpression with 13 genes. Among the interacted genes, 10 genes show interaction with all the three genes (PD-1, PD-L1, and PD-L2). Therefore, we enriched all of these genes in clusterProfiler. Finally, biological process (BP) analysis indicated that the interacted genes were mainly associated with mononuclear cell proliferation, regulation of mononuclear cell proliferation, leukocyte cell adhesion, and lymphocyte activation. KEGG pathway analysis enriched the interacted genes in pathways of cell differentiation of Th1, Th2, Th17, landscape of hematopoietic cells, and human T-cell leukemia virus 1 infection (Figure 4(b))(Table 2).

### 3.4. Association of PD Genes with Immune Cell Infiltration

**Figure 5:**
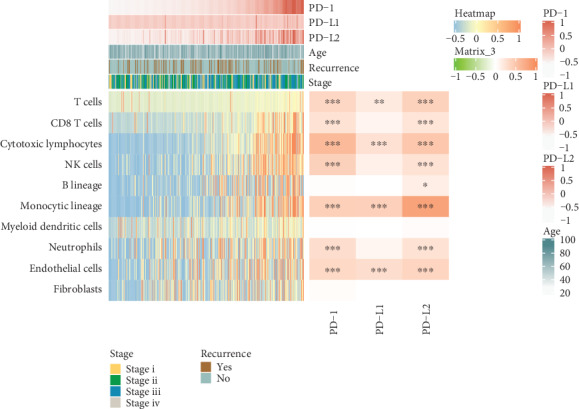


The landscape of various immune cell infiltration across different groups and stages of GC was visualized in Figure 5. Additionally, the middle heatmap in Figure 5 showed the relationship between PD genes and immune cell compositions on the basis of analysis of the RNA data. Resultly, PD-1 was mainly related with cytotoxic lymphocytes (*r* = 0.588) and endothelial cells (*r* = 0.401); PD-L1 were mainly related with monocytic lineage (*r* = 0.411); PD-L2 showed a significant correlation with myeloid dendritic cells (*r* = 0.800).

### 3.5. Association of PD Genes with Immune Checkpoints

**Figure 6:**
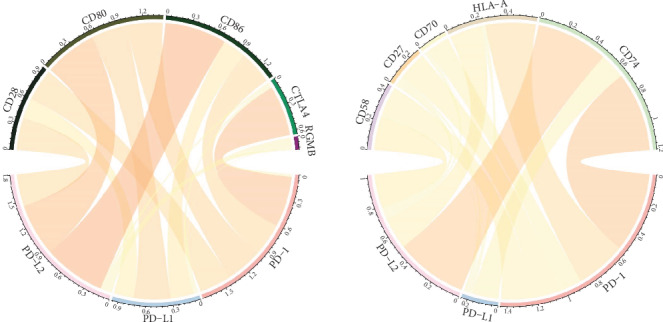


It has been found that core immune checkpoints including HLA-A, CD80, RGMB, CTLA4, CD58, CD86, CD27, CD70, CD28, and CD74 were implicated in the PD1/PD-L1/PDL2 regulatory axis. Relationship of PD gene expression with key immune checkpoints was subsequently investigated. As shown in Figure 6 and Table 3, PD-1, PD-L1, and PD-L2 closely associated with these critical immune checkpoints in GC: PD-1 was mainly related with CTLA4 (*r* = 0.826) and CD27 (*r* = 0.798); PD-L1 demonstrated significant association with CD80 (*r* = 0.812) and CD86 (*r* = 0.754); PD-L2 was significantly associated with CD86 (*r* = 0.922) and CD80 (*r* = 0.866).

## 4. Discussion

Immunotherapeutic agents have become an increasing promising tool for treatment of GC, as the immune system is the basal mechanism in humans to eliminate cancer. A great number of researchers have found that immune checkpoints such as PD-1/PD-L1/PD-L2 and cytotoxic T-lymphocyte-associated antigen-4 (CTLA-4) enable cancer cells to bypass human immunosurveillance, which therefore might be promising targets for immunotherapy. Previously, higher expressions of PD-1 and PD-L1 have been found to correlate with better prognosis of colorectal cancer patients based on TCGA database [17]. Similarly, a high PD-1 expression predicted better survival of breast cancer patients according to a study based on TCGA database [18]. In this study, we analyzed multiple information from TCGA to visualize the distributions of PD-1, PD-L1, and PD-L2 in relation to clinical parameters and survival of GC. In addition, association of somatic mutation, immune cell infiltration, and other essential immune factors with PD-1 axis was also investigated to stress the importance of PD-1 and the corresponding ligands in immune regulation of GC.

The analysis of PD-1/PD-L1/PD-L2 expression in different clinical groups suggested that PD-1/PD-L1/PD-L2 demonstrated no significant relationship with recurrence of GC. However, significant correlation between PD-L2 and clinical stage was observed. Survival analysis of GC patients showed that expression of PD-1 correlated with longer survival of GC patients. On contrary, expression of PD-L2 was significantly related with worse survival of GC. It has been revealed that PD-L1 expression demonstrate significant relation with age, stage, tumor size, invasion depth, lymph node metastasis, and venous invasion of GC [19]. A high expression of PD-L1 showed correlation with tumor invasion and unfavorable prognosis in GC [20]. In addition, one study of 240 GC patients suggested that positive PD-L1 expression on tumor-infiltrating lymphocytes predict worse overall survival than that with negative PD-L1 expression [21]. It has been reported that intratumoural expression of PD-L1 turns out to be a predictor of shorter survival of Epstein-Barr virus-related GC patients [22]. In a study assessing the prognostic value of PD-L1 mRNA expression in blood specimens of GC patients, significant association of PD-L1 expression and worse prognosis was observed [23]. Although several studies suggested that PD-L1 might be associated with the clinical outcome of GC patients, our analysis of TCGA data demonstrated that PD-1 and PD-L2 might be a prognostic marker for GC. The difference might due to the different expression level of mRNA and protein or the various examination methods of sequencing and traditional tools. The exact correlation between PD-1/PD-L1/PD-L2 expression and clinical outcome still require further studies to clarify.

We next analyzed mutation information of 290 GC individuals on the basis of TCGA data. The results suggested that although levels of PD-1/PD-L1/PD-L2 were not directly correlated to the total mutation load of each individual, mutations of PIK3CA could alter the expression of all PD genes. And TP53 demonstrated significant association with expression of PD-L1 and PD-L2. PI3K contributes to various biological functions including serine and threonine kinase AKT activation, which promotes the activation of mTOR [24]. The PI3K-Akt-mTOR axis is indispensable for modulation of cancer-related behaviors including cell vitality, proliferation, and cell cycle control; the mutations of which is commonly detected in tumor, thereby making it promising therapeutic targets [25]. As one of the most important tumor-suppressor, TP53 mutation has long been recognized as a factor for carcinogenesis [26]. Several researches have reported the positive correlation of TP53 mutation with PD-L1 expression in different types of cancers [27–29]. Our findings of the correlation between PIK3CA, TP53 mutations, and PD-L1 expression might provide novel insights into the mechanisms of PD-L1 modulation in cancer development.

Immune cell infiltration among tumor cells has been found to be closely implicated in the clinical outcome of tumor development. Our investigation of the association between PD genes and immune infiltration indicated that PD-1 was mainly related with cytotoxic lymphocytes and endothelial cells; PD-L1 were mainly related with monocytic lineage; PD-L2 showed significant correlation with myeloid dendritic cells. As for the influence of PD-1/PD-L1/PD-L2 on core immune factors including CD58, CD74, CD80, CD28, HLA-A, CD70, CD86, RGMB, CTLA4, CD27, and PD-1, they significantly correlated with CTLA4 and CD27; PD-L1 mainly correlated with CD80 and CD86; PD-L2 significantly correlated with CD86 and CD80. The PD-L1 level has been reported to correlate with increased densities of CD3-positive and CD8-positive tumor-infiltrating lymphocytes in GC patients [30]. PD-1 and TIM-3 could negatively modulate tumor antigen-specific CD8-positive T cells in human GC [31]. In addition, a close correlation between M2-like macrophage infiltration with PD-L1 expression in gastric adenocarcinoma was observed [32]. The potential complex interaction of PD genes with immune infiltration and other immune checkpoints might be an interesting research direction to improve the effect of clinical immune therapy.

After coexpression analysis, we finally obtain 831 PD-1 correlated genes, 1162 PD-L1 correlated genes, and 1997 genes interacting with PD-L2. We performed enrichment analysis of genes interacting with PD-1, PD-L1, and PD-L2. Finally, biological process analysis indicated terms of mononuclear cell proliferation, regulation of mononuclear cell proliferation, leukocyte cell-cell adhesion, and positive regulation of lymphocyte activation. KEGG pathway analysis enriched the interacted genes in pathways of differentiation of Th1, Th2, Th17, and hematopoietic cell landscape. Previously, miR-21 has been reported to contribute to the PD-1-PD-L1 axis-induced imbalance of Th17 and Treg cells in postoperative GC patients. The identified biological processes and pathways might contain valuable information of PD-1, PD-L1, and PD-L2 regulation in GC, which require further molecular investigations to clarify.

## 5. Conclusion

We characterized the distributions of PD-1/PD-L1/PD-L2 in relation to clinical parameters and survival of gastric cancer. Somatic mutation, immune cell infiltration, and other essential immune factors were closely implicated in the PD-1 axis. PD-1/PD-L1/PD-L2 coexpressed genes showed enrichment in mononuclear cell proliferation, leukocyte cell-cell adhesion, lymphocyte activation, and cell differentiation of Th1, Th2, and Th17. These findings might provide novel insights into the improvement of PD-1/PD-L1/PD-L2 immune therapy for gastric cancer patients.

## Figures and Tables

**Table 1:** Association of mutations with PD-1, PD-L1, and PD-L2 expressions in gastric cancer.

Gene	Mutation	PD-1			PD-L1			PD-L2		
		Mean	SD	*P*	Mean	SD	*P*	Mean	SD	*P*
ARID1A	No	0.612	1.164		2.407	23.912		0.508	1.133	
ARID1A	Yes	0.673	0.844	0.877	0.646	0.835	0.866	0.567	0.862	0.719
CSMD1	No	0.597	1.176		1.032	5.874		0.458	0.987	
CSMD1	Yes	0.705	1.046	0.480	9.621	58.396	0.203	0.791	1.682	0.281
CSMD3	No	0.582	1.101		2.848	27.095		0.494	0.971	
CSMD3	Yes	0.712	1.318	0.706	0.846	2.000	0.924	0.558	1.520	0.506
DNAH5	No	0.626	1.221		2.652	25.535		0.506	1.164	
DNAH5	Yes	0.540	0.660	0.724	0.667	1.315	0.797	0.527	0.877	0.892
FAT3	No	0.619	1.198		2.684	25.486		0.523	1.197	
FAT3	Yes	0.582	0.868	0.590	0.428	0.463	0.981	0.427	0.511	0.763
FAT4	No	0.655	1.250		2.718	26.117		0.529	1.194	
FAT4	Yes	0.429	0.544	0.722	0.784	1.614	0.870	0.420	0.747	0.919
FLG	No	0.649	1.256		2.439	25.698		0.540	1.237	
FLG	Yes	0.466	0.555	0.991	2.053	11.588	0.647	0.379	0.388	0.758
HMCN1	No	0.614	1.168		2.648	25.234		0.498	0.926	
HMCN1	Yes	0.611	1.085	0.669	0.368	0.442	0.419	0.591	2.062	0.159
LRP1B	No	0.640	1.254		2.865	26.803		0.544	1.223	
LRP1B	Yes	0.524	0.723	0.836	0.631	1.212	0.579	0.389	0.687	0.892
MUC16	No	0.580	1.186		1.155	6.308		0.499	1.064	
MUC16	Yes	0.704	1.072	0.767	5.652	44.389	0.619	0.538	1.286	0.874
OBSCN	No	0.603	1.188		2.683	25.641		0.539	1.210	
OBSCN	Yes	0.671	0.968	0.189	0.584	0.797	0.211	0.345	0.383	0.670
PCDH15	No	0.645	1.233		2.386	25.271		0.543	1.213	
PCDH15	Yes	0.456	0.625	0.450	2.255	12.515	0.541	0.341	0.467	0.111
PCLO	No	0.636	1.222		2.299	24.906		0.535	1.197	
PCLO	Yes	0.479	0.606	0.683	2.765	13.618	0.882	0.356	0.486	0.129
PIK3CA	No	0.520	0.925		0.946	5.834		0.425	0.936	
PIK3CA	Yes	1.113	1.913	<0.001	9.890	57.716	<0.001	0.956	1.778	<0.001
RYR2	No	0.623	1.193		2.653	25.587		0.517	1.171	
RYR2	Yes	0.561	0.924	0.825	0.708	1.333	0.131	0.467	0.827	0.689
SPTA1	No	0.643	1.230		2.724	25.691		0.543	1.211	
SPTA1	Yes	0.451	0.591	0.580	0.406	0.523	0.557	0.325	0.392	0.260
SYNE1	No	0.654	1.190		2.925	26.804		0.564	1.257	
SYNE1	Yes	0.473	1.027	0.137	0.424	0.570	0.415	0.321	0.380	0.081
TP53	No	0.680	1.295		3.179	27.936		0.601	1.307	
TP53	Yes	0.448	0.677	0.125	0.333	0.484	0.015	0.280	0.313	0.014
TTN	No	0.640	1.178		1.268	6.978		0.475	0.699	
TTN	Yes	0.576	1.128	0.054	3.918	35.803	0.230	0.558	1.542	0.088
ZFHX4	No	0.634	1.190		2.287	24.572		0.529	1.185	
ZFHX4	Yes	0.462	0.855	0.372	2.951	14.867	0.650	0.360	0.466	0.411

**Table 2:** GO and KEGG pathway enrichment analyses of PD-1, PD-L1, and PD-L2 coexpression genes in gastric cancer.

ID	Description	*P*	Adj. *P*	Count
hsa04658	Th1 and Th2 cell differentiation	2.23*E* − 20	9.82*E* − 19	11
hsa04659	Th17 cell differentiation	1.28*E* − 19	2.82*E* − 18	11
hsa04640	Hematopoietic cell lineage	1.36*E* − 17	1.99*E* − 16	10
hsa05166	Human T-cell leukemia virus 1 infection	4.35*E* − 16	4.78*E* − 15	11
hsa05169	Epstein-Barr virus infection	2.47*E* − 12	2.17*E* − 11	9
hsa05310	Asthma	3.70*E* − 12	2.71*E* − 11	6
hsa05140	Leishmaniasis	9.49*E* − 12	5.74*E* − 11	7
hsa04612	Antigen processing and presentation	1.04*E* − 11	5.74*E* − 11	7
hsa05330	Allograft rejection	1.38*E* − 11	6.74*E* − 11	6
hsa05332	Graft-versus-host disease	2.24*E* − 11	9.86*E* − 11	6
GO:0032943	Mononuclear cell proliferation	5.52*E* − 07	6.03*E* − 06	5
GO:0032944	Regulation of mononuclear cell proliferation	1.98*E* − 07	2.53*E* − 06	5
GO:0007159	Leukocyte cell-cell adhesion	1.01*E* − 09	2.13*E* − 08	7
GO:0051251	Positive regulation of lymphocyte activation	1.89*E* − 10	5.32*E* − 09	7
GO:1903039	Positive regulation of leukocyte cell-cell adhesion	5.08*E* − 11	1.65*E* − 09	7
GO:0019886	Antigen processing and presentation of exogenous peptide antigen	3.59*E* − 11	1.51*E* − 09	6
GO:0050870	Positive regulation of T cell activation	2.94*E* − 11	1.38*E* − 09	7
GO:0042110	T cell activation	2.09*E* − 12	1.47*E* − 10	9
GO:0060333	Interferon-gamma-mediated signaling pathway	1.16*E* − 13	9.75*E* − 12	7
GO:0050852	T cell receptor signaling pathway	9.42*E* − 24	3.98*E* − 21	12

**Table 3:** Association of PD-1, PD-L1, and PD-L2 with expression of key immune biomarkers.

Gene	PD-1			PD-L1			PD-L2		
	*r*	*P*	Adj. *P*	*r*	*P*	Adj. *P*	*r*	*P*	Adj. *P*
CD28	0.718	1.30*E* − 60	3.25*E* − 60	0.587	3.89*E* − 36	7.79*E* − 36	0.801	5.70*E* − 85	1.90*E* − 84
CD80	0.687	1.07*E* − 53	1.78*E* − 53	0.813	1.84*E* − 89	1.84*E* − 88	0.866	2.32*E* − 114	1.16*E* − 113
CD86	0.691	1.84*E* − 54	3.67*E* − 54	0.754	3.30*E* − 70	1.65*E* − 69	0.922	4.06*E* − 156	4.06*E* − 155
CTLA4	0.826	9.29*E* − 95	9.29*E* − 94	0.725	2.98*E* − 62	9.92*E* − 62	0.742	9.38*E* − 67	2.34*E* − 66
RGMB	0.290	1.09*E* − 08	1.21*E* − 08	0.307	1.28*E* − 09	1.28*E* − 09	0.451	3.77*E* − 20	4.18*E* − 20
CD58	0.258	4.25*E* − 07	4.25*E* − 07	0.516	6.43*E* − 27	1.07*E* − 26	0.480	5.15*E* − 23	6.44*E* − 23
CD27	0.798	6.40*E* − 84	3.20*E* − 83	0.509	3.87*E* − 26	5.53*E* − 26	0.685	2.53*E* − 53	4.21*E* − 53
CD70	0.563	1.08*E* − 32	1.54*E* − 32	0.499	5.83*E* − 25	7.29*E* − 25	0.553	1.84*E* − 31	2.64*E* − 31
HLA-A	0.509	4.39*E* − 26	5.49*E* − 26	0.483	2.79*E* − 23	3.10*E* − 23	0.409	1.50*E* − 16	1.50*E* − 16
CD74	0.735	7.96*E* − 65	2.65*E* − 64	0.617	9.63*E* − 41	2.41*E* − 40	0.691	1.53*E* − 54	3.05*E* − 54

## Data Availability

All the data used in the manuscript are freely available online.
